# ADVANCE CARE PLANNING: OUTCOMES FROM A TARGETED EDUCATIONAL INITIATIVE WITH OLDER SPANISH SPEAKERS

**DOI:** 10.1093/geroni/igae098.2380

**Published:** 2024-12-31

**Authors:** Susanny Beltran

**Affiliations:** University of Central Florida, Orlando, Florida, United States

## Abstract

Targeted interventions are essential to enhance knowledge and engagement in end-of-life care among Spanish-speaking older adults, to address existing disparities in advance care planning (ACP). This study evaluated the effectiveness of a resource-efficient, culturally tailored educational intervention in improving ACP readiness and knowledge among Spanish-speaking older adults. A quasi-experimental pretest-posttest design was used to assess the impact of an intervention involving an educational presentation on topics such as ACP, end-of-life care options including curative and palliative models of care and sharing relevant resources. The study involved community-dwelling older Latinos (aged 61-94) in a southern U.S. state who were engaged with adult day care centers. Measures included participants’ knowledge of ACP, care options, familiarity with hospice and palliative care, and attitudes toward hospice, assessed using pre- and post-intervention surveys. Statistically significant improvements were observed in ACP knowledge, understanding of care options, and attitudes towards hospice and palliative care post-intervention. Age, marital status, and education level influenced knowledge scores, with no significant gender differences in the intervention’s efficacy. The educational intervention effectively enhanced end-of-life care planning readiness and knowledge among older Latinos. The study highlights the potential for sustainable, accessible, and culturally sensitive educational strategies to reduce disparities in ACP knowledge and possibly engagement. Future research will explore the feasibility of training laypersons and adult day care staff to deliver the educational presentation.

